# The impact of gum-chewing on postoperative ileus following gynecological cancer surgery: A systematic review and meta-analysis of randomized controlled trials

**DOI:** 10.3389/fonc.2022.1059924

**Published:** 2023-01-17

**Authors:** Ya-Nan Yin, Hong Xie, Jian-Hua Ren, Ni-Jie Jiang, Li Dai

**Affiliations:** ^1^ Department of Gynecology and Obstetrics, West China Second University Hospital, Sichuan University, Chengdu, China; ^2^ West China School of Nursing, Sichuan University, Chengdu, China; ^3^ Key Laboratory of Birth Defects and Related Diseases of Women and Children, Ministry of Education, Sichuan University, Chengdu, China; ^4^ National Center for Birth Defects Monitoring, West China Second University Hospital, Sichuan University, Sichuan, China

**Keywords:** gastrointestinal function, gum-chewing, gynecological cancer surgery, meta-analysis, review

## Abstract

**Objective:**

To assess the effect and safety of gum-chewing on the prevention of postoperative ileus after gynecological cancer surgery.

**Methods:**

We conducted a systematic review of randomized controlled trials (RCTs) published between 2000 and 2022 in English and Chinese, using the EBSCO, Web of Science, Scopus, Cochrane Central Register of Controlled Trials (Cochrane database), PubMed, Medline (via Ovid), Chinese National Knowledge Infrastructure (CNKI), China Science and Technology Journal Database, and Wan Fang databases. A total of 837 studies were screened using Endnote software, and those that met the inclusion criteria were selected for analysis. The main outcome of interest was the incidence of postoperative ileus, and secondary outcomes included time to first flatus, time to first bowel movement, and length of hospital stay.

**Results:**

Two authors extracted data and performed quality assessment independently. The review included six RCTs with a total of 669 patients. Compared with routine care, gum-chewing could significantly reduce the incidence of postoperative ileus (RR 0.46, 95% CI: 0.30, 0.72, P=0.0006), shorten the time to first flatus (WMD -9.58, 95% CI: -15.04, -4.12, *P*=0.0006), first bowel movement (WMD -11.31, 95% CI: -21.05, -1.56, *P*=0.02), and the length of hospital stay (WMD -1.53, 95% CI: -2.08, -0.98, *P*<0.00001).

**Conclusions:**

Gum-chewing is associated with early recovery of gastrointestinal function after gynecological cancer surgery and may be an effective and harmless intervention to prevent postoperative ileus.

**Systemaic review registration:**

https://www.crd.york.ac.uk/prospero/#searchadvanced, identifier CRD42022384346.

## 1 Introduction

Cancer is the leading cause of death worldwide and has become a major public health concern (1). Gynecological cancers are malignant tumors that affect the female reproductive system and account for more than 16% of all cancers among women (2). Globally, the top three most common gynecological cancers are cervical, ovarian, and uterine cancers. In the United States alone, approximately 109,000 new cases of these cancers were diagnosed in 2019, and there are an estimated 33,100 deaths from these diseases each year (3).

Surgery to treat cancer can help alleviate symptoms and extend a patient’s life, it may also result in a slower recovery of bowel function and cause postoperative complications such as nausea, vomiting, abdominal pain, abdominal distention, and even intestinal obstruction (4). Postoperative ileus is a temporary disruption of normal gastrointestinal function that is characterized by the inability to pass gas or stool due to non-mechanical causes (5). Despite the lack of a consistent definition, the incidence of postoperative ileus after abdominal surgery ranges from 5% to 25%, with an average incidence of 10% to 15% (6–8). The incidence of postoperative ileus in individuals receiving complete staging surgery for gynecologic cancers may be as high as 50% (9, 10). Postoperative ileus is reported to increase hospital costs by more than $5000 per patient and extend hospital stays by up to 5 days in the United States (11, 12). Therefore, postoperative ileus has a significant socio-economic impact due to the prolonged hospital stay and high overall hospital costs (13, 14).

Controversial theories exist about the cause of postoperative ileus. The development of postoperative ileus has been linked to sympathetic hyperactivity, pain fiber activation, and elevated catecholamine concentrations in the blood (15, 16). Another theory for the cause of postoperative ileus is aberrant electrolyte levels (17). Moreover, bowel manipulation-related pacemaker malfunction may possibly be another factor in postoperative ileus (18). Enhanced Recovery After Surgery (ERAS) is known to accelerate the motility of the gastrointestinal tract, and several interventions have been recommended to prevent postoperative ileus, including early mobilization, gum chewing, and minimally invasive surgery (19–21).

Previous research has shown that gum chewing is a form of sham feeding that can be used to restore gastrointestinal function without complications (22). It is believed that gum chewing may activate the cephalic-vagal axis, which can lead to the secretion of more gastrointestinal hormones (23). This, in turn, may help to prevent postoperative ileus by reducing inflammation of the intestinal wall (24). Gum chewing is a beneficial intervention that should be encouraged as a regular practice following gynecological surgery, based on several systematic reviews with meta-analyses (4, 25). One obvious limitation of these studies was their significant heterogeneity, and the majority of the participants in the trials were patients with benign gynecological diseases. Unlike benign gynecological surgery, gynecological cancer surgery often involves a larger abdominal incision and carries a higher risk of postoperative ileus (26). However, these findings are inconclusive, as there are some studies that suggest gum chewing may not promote gastrointestinal recovery after laparoscopic surgery (27–31), and there is no robust evidence to support the idea that gum chewing can improve the gastrointestinal function recovery for patients undergoing gynecological cancer surgery.

The aim of this study is to review randomized controlled trials that assess the effectiveness and safety of gum chewing for preventing postoperative ileus after gynecological cancer surgery. In addition to the incidence of postoperative ileus, which is the primary outcome of interest, the secondary outcomes include time to first flatus, time to first bowel movement, time to first defecation, and length of hospital stay.

## 2 Materials and methods

### 2.1 Search strategy

A comprehensive literature search for all RCTs was carried out between January 2000 and December 2022 in English and Chinese in EBSCO, Web of Science, Scopus, Cochrane Central Register of Controlled Trials (Cochrane database), PubMed, Medline (via Ovid), Chinese National Knowledge Infrastructure (CNKI), China Science and Technology Journal Database, and Wan Fang. We combined the terms (“gynecological surgery” OR “gynecologic surgery” OR “gynecologic surgical procedures” OR “gynecologic surgical procedure” OR “gynecological surgical procedure” OR “gynecological surgical procedures” OR “complete staging surgery” OR “comprehensive staging surgery” OR “modified hysterectomy” OR “salpingo-oophorectomy” OR “radical hysterectomy” OR “hysterectomy” OR “laparotomy” OR “adnexal surgery” OR “uterine surgery”) AND (“gum chewing” OR “chewing gum” OR “gum” OR “gums” OR “sham feeding”) and snowball searches of all included studies were carried out to ensure full inclusion. The Preferred Reporting Items for Systematic Reviews and Meta-Analysis (PRISMA) statement (32) was followed in conducting this systematic review and meta-analysis. Our protocol has been registered in the International prospective register of systematic reviews (PROSPERO, https://www.crd.york.ac.uk/prospero/#joinuppage), the registration number is CRD42022384346.

### 2.2 Study selection

A total of 837 studies were reviewed by Endnote software, with two review authors completing preliminary exclusions based on title and abstract review and then through full-text review respectively. We selected the inclusion criteria according to the PICOS, patients (P) were subjects who received gynecological cancer surgery, interventions (I) were routine care with gum-chewing, comparison (C) was only routine care, outcomes (O) should include no less than one of the indicators below: incidence of postoperative ileus, time to first flatus, time to first bowel movement, or length of hospital stay, and study design (S) was limited to randomized controlled trials (RCTs). Papers were excluded if the full text was not available, published in a foreign language (except English and Chinese), and studies were conducted in children. Discussions or consultations with a third party (RJH) were used to settle disagreements between the two review writers.

### 2.3 Data extraction

A data extraction form was designed for all of the included articles, two authors (YYN and XH) independently assessed and extracted the following information: Author/year, country, sample size, type of surgery, methods of gum chewing, the definition of postoperative ileus, complication, and main outcomes. We resolved the differences between the two reviewers through consensus discussions with a third person on the review team (RJH) when necessary. When research data were not presented in the article, we contacted the authors of the original study to ask for the data. The data presented as “median and range” values were computed to mean and standard deviation (SD) data with the equations described by Wan et al. (33) and Luo et al. (34) to ensure that all of the relevant data could be included in the final quantitative synthesis.

### 2.4 Quality assessment of the included studies

In order to evaluate methodological quality and risk of bias in RCTs, our study adopted the Cochrane Collaboration’s “risk of bias” instrument (35). The following areas were evaluated in this analysis: representativeness of study population, random sequence generation, blinding of outcome assessors and participants, allocation concealment, selective reporting of outcomes, incomplete outcome data, and other potential sources of bias. Based on predetermined criteria, each of these domain was categorized as having a high, unclear, or low risk of bias.

### 2.5 Data synthesis and analysis

Review Manager (RevMan) version 5.3 software was used to format the extracted data for meta-analysis. Effect estimates were presented as weighted mean differences (WMDs) for continuous outcomes and risk ratios (RRs) for dichotomous data with 95% confidence intervals (95% CIs). For continuous data and dichotomous variables, the inverse variance estimate method and the Mantel-Haenzsel method, respectively, were used. The Higgins’ *I^2^* statistic was used to determine the degree of statistical heterogeneity (*I^2^ ≤* 25%, 25%<*I^2^ ≤* 75%, and *I^2^* >75 represent the low, moderate, and high heterogeneity, respectively) (36). Given the variability in the definition of postoperative ileus and the different surgical approaches (laparotomy versus laparoscopy), it was decided that a random effects model would be most appropriate for this meta-analysis (37). Subgroup analysis was presented if heterogeneity was observed among the studies. We used STATA software (Version 14.0) (Stata Corp., College Station, Texas) to perform sensitivity analysis and potential publication bias. Qualitative analysis was conducted if the heterogeneity was large.

## 3 Results

### 3.1 Characteristics of included studies

**Figure 1:**
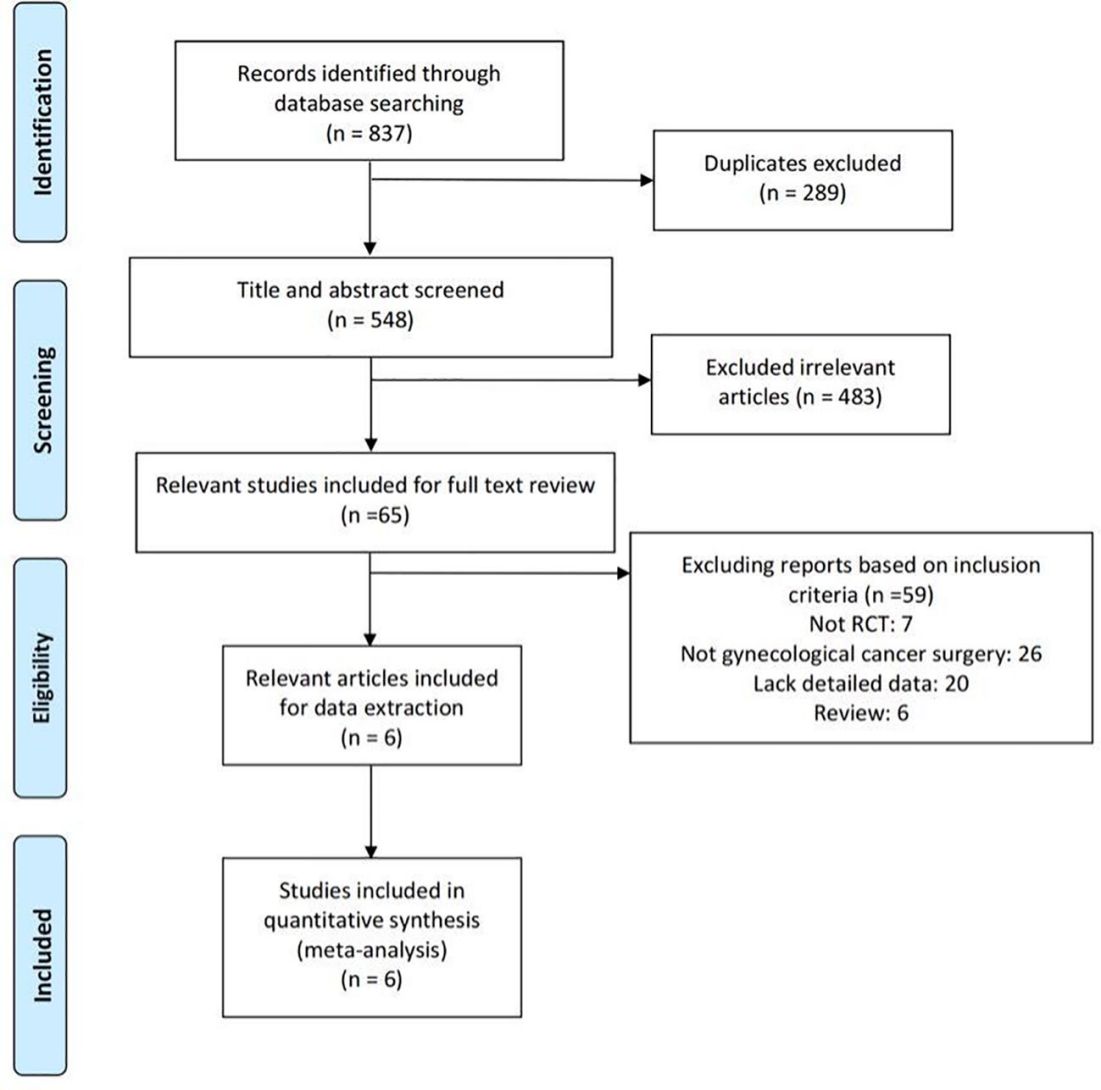
The flowchart of search result.

The literature search and selection are illustrated in the PRISMA flow diagram (**Figure 1**). The search approach initially turned up 837 pertinent publications in total (**Table 1**). Of these, 289 articles were excluded due to duplicates, and 483 trials were excluded after being screened based on their titles and abstracts. After two review authors re-viewed the full text, an additional 59 reports were excluded because they did not meet the inclusion criteria. The quantitative synthesis was completed with the inclusion of six research (3, 38–42).

**Table 1:** Search terms and results.

Database	Search strategy	Results
Web of science	(TS=(“gynecological surgery” OR “gynecologic surgery” OR “gynecologic surgical procedures” OR “gynecologic surgical procedure” OR “gynecological surgical procedure” OR “gynecological surgical procedures” OR “complete staging surgery” OR “comprehensive staging surgery” OR “modified hysterectomy” OR “salpingo-oophorectomy” OR “radical hysterectomy” OR “hysterectomy” OR “laparotomy” OR “adnexal surgery” OR “uterine surgery”) ) AND (TS=(“gum chewing” OR “chewing gum” OR “gum” OR “gums” OR “sham feeding”))	61
Scopus	ALL(“gynecological surgery” OR “gynecologic surgery” OR “gynecologic surgical procedures” OR “gynecologic surgical procedure” OR “gynecological surgical procedure” OR “gynecological surgical procedures” OR “complete staging surgery” OR “comprehensive staging surgery” OR “modified hysterectomy” OR “salpingo-oophorectomy” OR “radical hysterectomy” OR “hysterectomy” OR “laparotomy” OR “adnexal surgery” OR “uterine surgery”) AND ABS(“gum chewing” OR “chewing gum” OR “gum” OR “gums” OR “sham feeding”) AND PUBYEAR AFT 2000	108
Cochrane Central Register of Controlled Trials (Cochrane database)	ALL Text ((“gynecological surgery” OR “gynecologic surgery” OR “gynecologic surgical procedures” OR “gynecologic surgical procedure” OR “gynecological surgical procedure” OR “gynecological surgical procedures” OR “complete staging surgery” OR “comprehensive staging surgery” OR “modified hysterectomy” OR “salpingo-oophorectomy” OR “radical hysterectomy” OR “hysterectomy” OR “laparotomy” OR “adnexal surgery” OR “uterine surgery”) AND (“gum chewing” OR “chewing gum” OR “gum” OR “gums” OR “sham feeding”))	53
EBSCO	TX (“gynecological surgery” OR “gynecologic surgery” OR “gynecologic surgical procedures” OR “gynecologic surgical procedure” OR “gynecological surgical procedure” OR “gynecological surgical procedures” OR “complete staging surgery” OR “comprehensive staging surgery” OR “modified hysterectomy” OR “salpingo-oophorectomy” OR “radical hysterectomy” OR “hysterectomy” OR “laparotomy” OR “adnexal surgery” OR “uterine surgery”) AND SU (“gum chewing” OR “chewing gum” OR “gum” OR “gums” OR “sham feeding”)	68
Pubmed	(“Chewing Gum”[MeSH Terms] OR (“gum chewing”[All Fields] OR “gum”[All Fields] OR “gums”[All Fields] OR “sham feeding”[All Fields])) AND (“Gynecologic Surgical Procedures”[MeSH Terms] OR “hysterectomy”[MeSH Terms] OR “salpingo oophorectomy”[MeSH Terms] OR (“gynecological surgery”[All Fields] OR “gynecologic surgery”[All Fields] OR “gynecologic surgical procedure”[All Fields] OR “gynecological surgical procedure”[All Fields] OR “gynecological surgical procedures”[All Fields] OR “complete staging surgery”[All Fields] OR “comprehensive staging surgery”[All Fields] OR “modified hysterectomy”[All Fields] OR “radical hysterectomy”[All Fields] OR “laparotomy”[All Fields] OR “adnexal surgery”[All Fields] OR “uterine surgery”[All Fields]))	64
Medline (via Ovid)	(gynecological surgery or gynecologic surgery or gynecologic surgical procedures or gynecologic surgical procedure or gynecological surgical procedure or gynecological surgical procedures or complete staging surgery or comprehensive staging surgery or modified hysterectomy or salpingo-oophorectomy or radical hysterectomy or hysterectomy or laparotomy or adnexal surgery or uterine surgery).af. AND (gum chewing or chewing gum or gum or gums or sham feeding).af. AND limit 3 to yr=”2000-Current”	44
CNKI	FT = (“gum chewing” OR “gum” OR “sham feeding”)) AND FT = (“gynecological surgery” OR “complete staging surgery” OR “modified hysterectomy” OR “salpingo-oophorectomy” OR “radical hysterectomy” OR “hysterectomy” OR “laparotomy” OR “adnexal surgery” OR “uterine surgery”))	299
Wan Fang	(TS: (“gum chewing” OR “gum” OR “sham feeding”)) AND (TS: (“gynecological surgery” OR “complete staging surgery” OR “comprehensive staging surgery” OR “modified hysterectomy” OR “salpingo-oophorectomy” OR “radical hysterectomy” OR “hysterectomy” OR “laparotomy” OR “adnexal surgery” OR “uterine surgery”))	49
China Science and Technology Journal Database	(M=(“gum chewing” OR “chewing gum” OR “gum” OR “gums” OR “sham feeding”)) AND (M = (“gynecological surgery” OR “complete staging surgery” OR “comprehensive staging surgery” OR “modified hysterectomy” OR “salpingo-oophorectomy” OR “radical hysterectomy” OR “hysterectomy” OR “laparotomy” OR “adnexal surgery” OR “uterine surgery”))	91

SU, subject; TS, topic; ABS, abstract; SU, research area; FT, full text; TX, text; M, title or keyword, CNKI, Chinese National Knowledge Infrastructure.

**Table 2** presents the fundamental traits of the six included trials, and **Table 3** summarizes the results. In brief, a total of 669 patients with gynecological cancer had surgery, and patients were randomly assigned to either routine postoperative care groups (n = 336) or gum-chewing intervention groups (n = 333). The six trials were conducted in three different countries. No complications were reported by any of the participants.

**Table 2:** Characteristics of gum chewing intervention in the six included randomized controlled studies.

Author/year,	Country	Sample size	Type of surgery	Interventions of gum chewing	Definition of postoperative ileus	Main outcome	Complication
Ertas et al, 2013 (40)	Turkey	149	Laparotomy	Chewing sugar-free gum 3 times a day for 30 minutes starting from the first postoperative morning	“Mild”: if ileus symptoms spontaneously resolved in a few days with observation and basic support, “moderate” if vomiting was persisted and a nasogastric tube re-insertion was necessary, and “severe” if symptoms persisted for more than two days or resisted previous treatment	①②③④	None
Huang G K et al, 2016 (42)	China	104	Laparotomy	Chewing gum 3 times a day for 30 minutes starting from the first postoperative morning	Self-report	①②③④	None
Li W et al, 2016 (41)	China	156	Laparotomy	Chewing sugar-free gum 3 times a day for 30 minutes starting from the first postoperative morning	Abdominal distension, absence of bowel movement/flatus/defecation over 24 h, two or more episodes of nausea or vomiting, and the amount of vomiting is greater than 100 ml	①②③④	None
Chen L 2017 (38)	China	78	Laparotomy	Chewing gum 3 times a day for 30 minutes starting from the first postoperative morning	Self-report	①②③④	None
Wu L M 2020 (39)	China	100	Laparotomy or laparoscopy	Chewing sugar-free gum 3 times a day for 30 minutes starting from the first postoperative morning	Self-report	①③④	None
Nanthiphatthanachai et al, 2020 *(* 3)	Thailand	82	Laparotomy or laparoscopy	Chewing sugar-free gum for 30 minutes starting on the first postoperative morning then every 8 hours and continue to the first passage of flatus	“Mild”: if ileus symptoms resolved spontaneously within a few days with only observation and basic support, “moderate” if vomiting persisted and reinsertion of the nasogastric tube was required, and “severe” if symptoms persisted for greater than two days or resisted treatment	①②③④	None

①postoperative ileus, ②time to first flatus, ③time to first bowel movement, ④length of hospital stay.

**Table 3:** Summary of outcomes across all included studies.

Study	Postoperative ileus incidence (N)					Time to first flatus (h)			Time to first bowel movement (h)			Length of hospital stay (d)		
	Intervention			Control		Intervention		Control	Intervention		Control	Intervention		Control
	N (%)	Total		N (%)	Total	Mean ± SD		Mean ± SD	Mean ± SD		Mean ± SD	Mean ± SD		Mean ± SD
Ertas 2013 (40)	12 (16.22)	74		37 (49.33)	75	34.0±11.5		43.6±14.0	41.5±15.7		50.1±15.9	5.9±1.1		7.0±1.4
Huang G K 2016 (42)	0 (0)	52		1 (1.92)	52	25.46±3.08		28.50±3.24	37.62±3.77		39.81±3.63	11.29±0.69		13.54±0.73
Li W 2016 (41)	2 (2.56)	78		5 (6.41)	78	41.2±10.3		56.3±11.5	20.8±9.9		28.5±10.6	6.5±0.7		7.7±1.2
Chen L 2017 (38)	2 (5.13)	39		4 (10.26)	39	23.6±3.7		35.4±4.2	37.6±5.2		58.4±5.8	2.9±1.2		4.6±1.5
Wu L M 2020 (39)	1 (2.00)	50		7 (14.00)	50	–		–	45.0±4.6		72.2±4.8	3.2±0.8		4.2±1.6
Nanthiphatthana-chai 2020 (3)	14 (35.00)	40		20 (47.62)	42	28.7±19.6		37.1±16.9	4.8±2.3		6.1±4.6	3.38±1.81		7.86±11.1

### 3.2 Methodological quality

**Figure 2:**
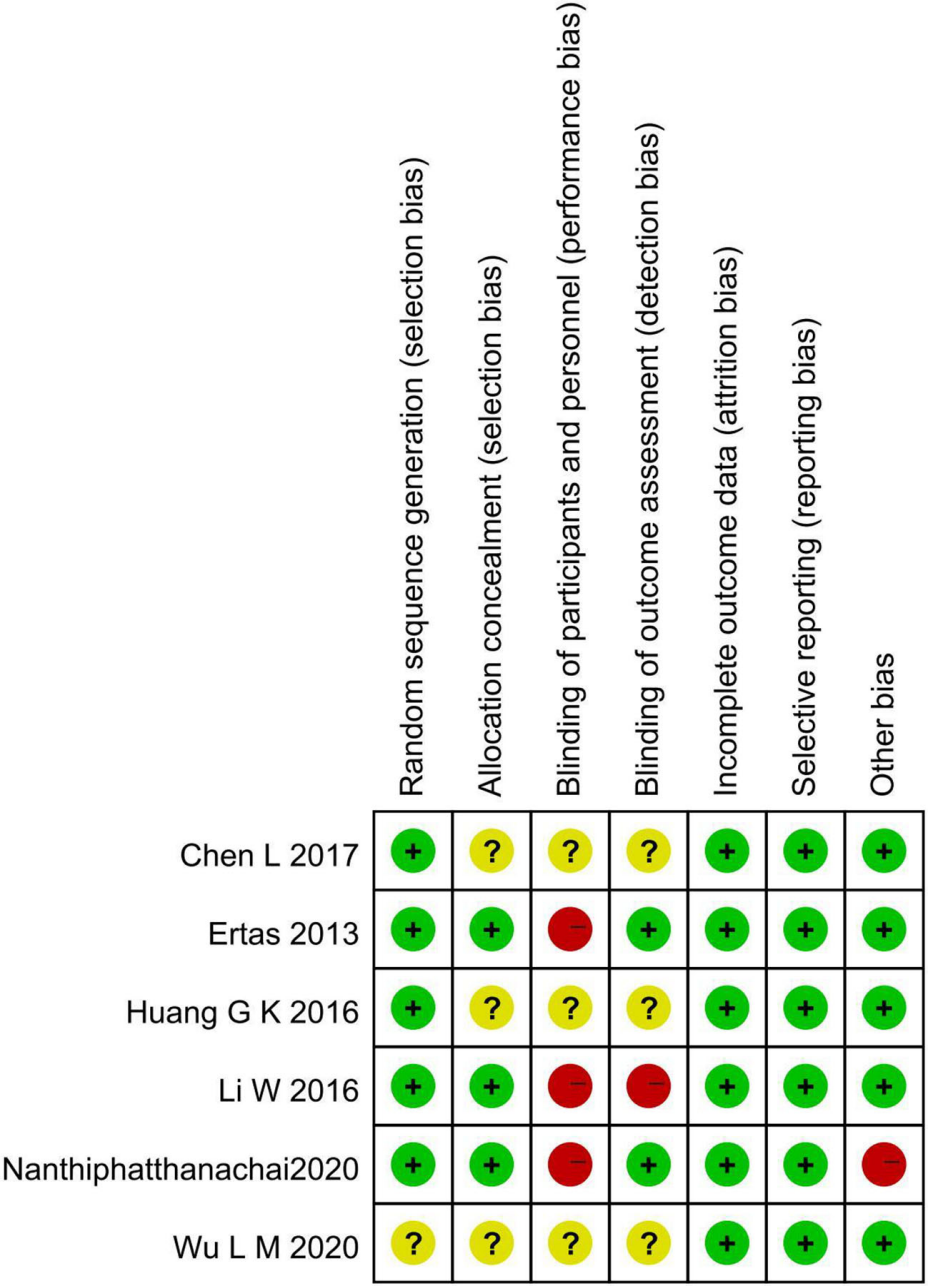
Methodological quality and risk of included trials.

Cross-tabulation in **Figure 2** shows the risk of bias for the included articles as determined by the Cochrane Risk of Bias Tool. While Chen L (38), Wu L M (39), and Huang G K 2016 (42) did not indicate the specific method of allocation concealment, three other included trials provided the right methods of random sequence generation and allocation concealment. Two studies chose to blind the outcome assessors (3, 40), and all trials did not blind subjects or not mentioned. There were no instances of selective or incomplete result reporting. The baseline was imbalanced in Nanthiphatthanachai 2020 (3), and no other obvious risk of potential bias was found in the included articles.

### 3.3 Primary outcome

**Figure 3:**
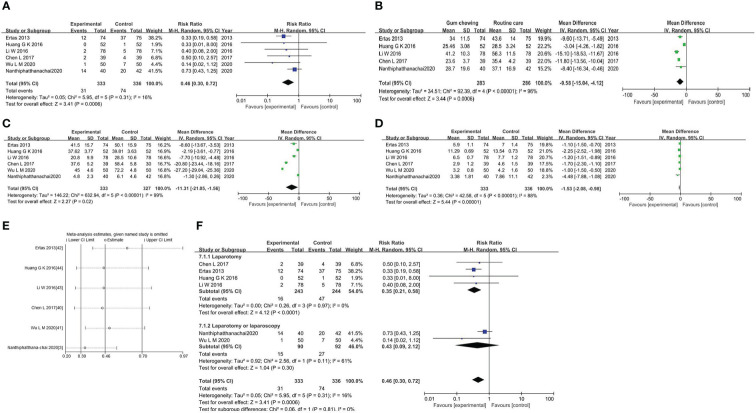
**(A)** Forest plot for the incidence of postoperative ileus. **(B)** Forest plot for the time to first flatus. **(C)** Forest plot for the time to first bowel movement. **(D)** Forest plot for the length of hospitalization. **(E)** Sensitivity analysis in studies assessing the impact of gum chewing. **(F)** Forest plot for the subgroup analysis.

The meta-analysis of the incidence of postoperative ileus was conducted on all trials involving 669 individuals. The results showed that the gum-chewing group had a significantly lower incidence of postoperative ileus, with an RR of 0.46 (95% CI: 0.30, 0.72, *P*=0.0006), and moderate level of heterogeneity (*I^2 ^* =16%) (**Figure 3A**).

### 3.4 Secondary outcomes

The time to first flatus in the gum-chewing group was significantly shorter than that in the routine care group. The WMD and *I^2^* values were -9.58 (95% CI: -15.04, -4.12, *P*=0.0006) and *I^2 ^=*96% (**Figure 3B**). Time to the first bowel movement in the gum-chewing group was significantly shorter than that in the routine care group. The WMD and *I^2^* values were -11.31 (95% CI: -21.05, -1.56, *P*=0.02) and *I^2 =^* 99% (**Figure 3C**). The length of hospitalization was significantly shorter in the gum-chewing group compared to the routine care group. The WMD and *I^2^* values were -1.53 (95% CI: -2.08, -0.98, *P*<0.00001) and *I^2 =^* 88% (**Figure 3D**).

Since the heterogeneity of secondary outcomes was large, we performed a qualitative analysis (**Table 4**). Only Nanthiphatthanachai 2020 (3) was conducted with the implementation of ERAS, the main cancer types of patients in Huang G K 2016 (42) were different from others. The measurement method was described in two studies (3, 40), and only Ertas 2013 (40) mentioned the same surgical team performing all operations while others did not. Criteria for hospital discharge were only reported in Ertas 2013 (40) and Nanthiphatthanachai 2020 (3).

**Table 4:** Qualitative analysis of the secondary outcomes in the included studies.

Author, year	Implementation of ERAS	Main cancer types of patients	Description of measurement method	Same surgical team	Criteria for hospital discharge
Ertas 2013 (40)	No	Ovarian cancer, endometrial cancer, and cervical cancer	Yes	Yes	Yes
Huang G K 2016 (42)	No	Ovarian cancer, endometrial cancer, and carcinoma of the fallopian tube	No	No	No
Li W 2016 (41)	No	Ovarian cancer, endometrial cancer, and cervical cancer	No	No	No
Chen L 2017 (38)	No	Ovarian cancer, endometrial cancer, and cervical cancer	No	No	No
Wu L M 2020 (39)	No	Ovarian cancer, endometrial cancer, and cervical cancer	No	No	No
Nanthiphatthana-chai 2020 (3)	Yes	Ovarian cancer, endometrial cancer, and cervical cancer	Yes	No	Yes

ERAS, Enhanced recovery after surgery.

### 3.5 Sensitivity analysis and subgroup analysis

We performed a sensitivity analysis by sequentially removing each study with STATA software, and the results indicated a consistent result among all studies (**Figure 3E**). The subgroup analysis based on the type of surgery showed that gum chewing in patients who underwent laparotomy had a lower incidence of postoperative ileus by an RR of 0.35 (95% CI: 0.21, 0.58, *P*<0.0001), and the heterogeneity was low with an *I^2^* value of 0%. No statistically significant difference was observed among patients who underwent laparotomy or laparoscopy in two groups by an RR of 0.43 (95% CI: 0.09, 2.12, *P*=0.30), and heterogeneity among the studies was medium (*I^2 =^* 61%) (**Figure 3F**).

### 3.6 Publication bias analysis

**Figure 4:**
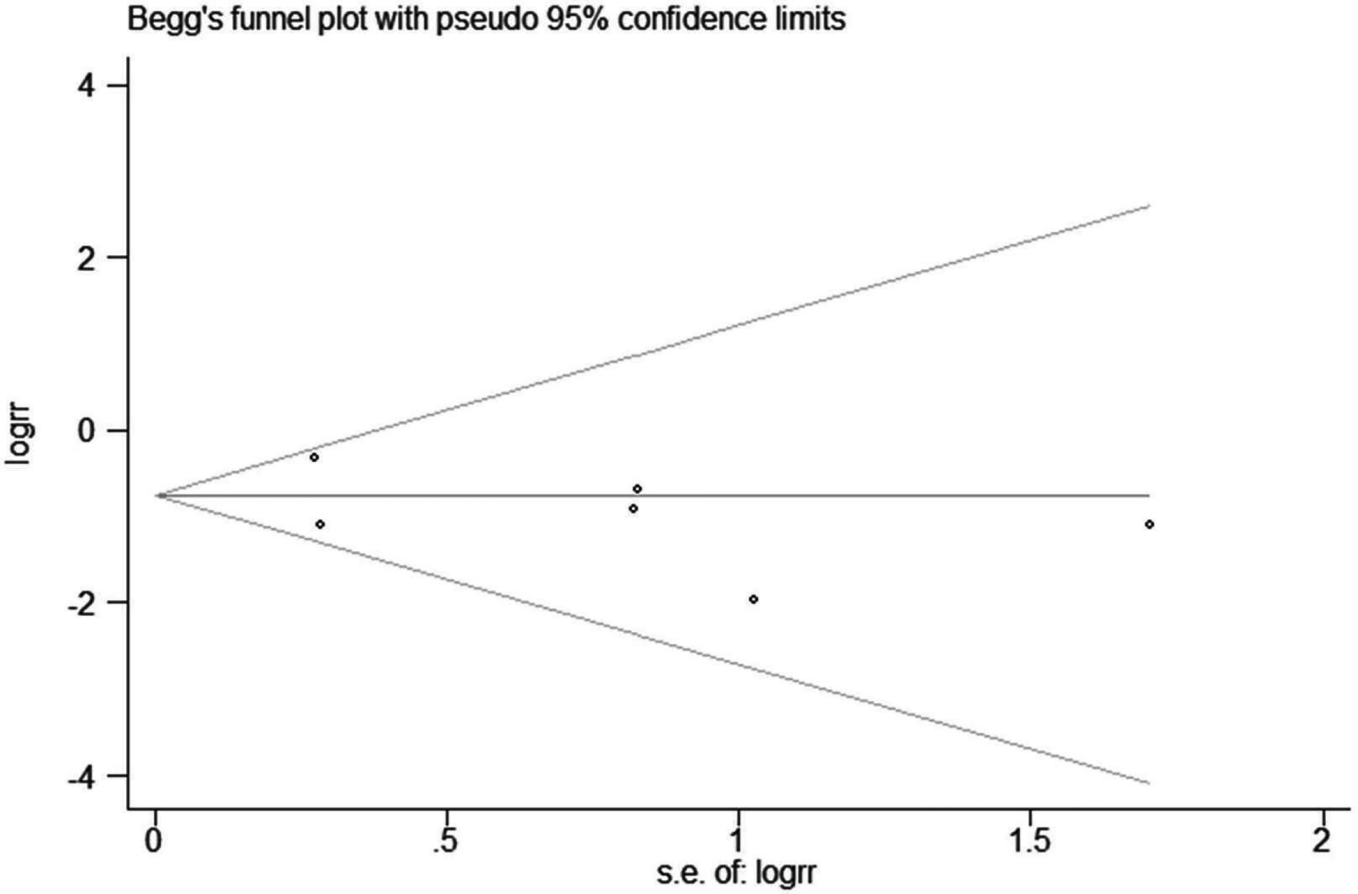
Begg’s funnel plot for the incidence of postoperative ileus.

Funnel plots are commonly used to detect publication bias (36). We performed funnel plot analysis of the incidence of postoperative ileus (**Figure 4**). The potential publication bias was assessed with the Egger’s and Begg’s tests, and no significant potential publication bias was observed (*P >*0.05).

## 4 Discussion

### 4.1 Quality of the evidence

Based on evidence from six RCTs involving 669 participants, we found that gum chewing after gynecological cancer surgery played an important role in reducing the incidence of postoperative ileus, shortening the time to first flatus, first bowel movement, and length of hospitalization. No complications were reported in the included trials. The method of allocation concealment was not revealed in three trials (38, 39, 42), which may lead to a risk of selection bias. Because the nature of the study does not permit complete blindness in both participants and outcome assessors, the risk of performance bias cannot be completely avoided. Most of the studies included in this analysis are single-blinded for outcome assessors (3, 40), which may introduce the possibility of a placebo effect. In addition, there was an imbalance in the underlying diseases between the two study groups in Nanthiphatthanachai 2020 (3).

### 4.2 Implications

In recent years, there has been increasing evidence that gum-chewing following abdominal surgery contributes to improved postoperative intestinal function in patients (43, 44). Dutch researchers found that 27% of participants in the gum-chewing group developed postoperative ileus after surgery, compared to 48% of participants in the control group, and they also found that gum-chewing was associated with a reduction of inflammatory markers (45). In a meta-analysis of gynecological surgery, Xu and his co-authors revealed that gum-chewing was an effective way to improve gastrointestinal function and reduce complications (4). These findings all concluded that gum-chewing was a novel, harmless strategy that should be advocated as a part of enhanced recovery after surgery (ERAS) protocol in patients who had abdominal surgery. Compared with our review, however, only two of the ten included trials where participants underwent gynecological cancer surgery in Xu’s study (4), and significant heterogeneity could not be avoided. The effect of gum-chewing in abdominal surgery was also inconclusive between studies. A multicenter randomized controlled trial (RCT) of 2000 patients undergoing abdominal surgery showed no differences in time to first flatus and time to defecation in both groups, and gum-chewing did not reduce hospital stays and postoperative complications (46). These findings were similar to previous studies (47, 48). These conflicting results suggest that caution should be taken when evaluating the effectiveness of gum-chewing after abdominal surgery, and further evidence should be gathered in future studies.

For gynecologic/oncology surgery, the enhanced recovery after surgery (ERAS) guidelines were developed in 2016 for postoperative care and has been proved to have many advantages, such as preventing postoperative ileus and accelerating bowel function (49). However, these mandatory requirements such as gum-chewing are not routinely implemented due to low recommendation grade and limited clinical benefits. One trial (3) of our review was conducted in relation to the implementation of ERAS, which may be a confounder, thus ERAS should be considered as an independent factor in future research. Other confounding factors that may affect outcomes include outcome indicators, type of surgery, and the definition of postoperative ileus. The first passage of flatus after surgery, a commonly reported outcome, is a signal that patients are recovering from the surgery and are no longer at risk of ileus (25). However, the measurement of this indicator is too subjective and lacks accuracy, since the data are derived from patients’ self-reports (50). This may explain the uncertain results and high heterogeneity found in previous meta-analyses. Postoperative ileus occurs after almost all types of surgery. However, bowel motility in laparoscopic surgery is not like in laparotomy surgery (51, 52). Since muscle function progressively decreases with increasing surgical manipulation of the bowel, and in laparoscopic surgery the bowel is only minimally manipulated (53). Of the six trials included in this study, two trials involved both laparoscopic and laparotomy surgery (3, 39), while the other four only involved laparotomy surgery (38, 40–42). The subgroup analysis based on the type of surgery showed that gum chewing in patients who underwent laparotomy had a lower incidence of postoperative ileus. No statistically significant difference was observed between the laparotomy and laparoscopy groups, indicating the type of surgery is a confounding factor.Additionally, the varying definition of postoperative ileus may also contribute to the heterogeneity. We found similar definitions reported in two studies (3, 40), which may reduce the potential for reporting bias. Similar gum-chewing interventions in the studies also contributed to the low heterogeneity. The main cancer types of patients and criteria for hospital discharge may also influence the heterogeneity. The main cancer types were ovarian cancer, endometrial cancer, and cervical cancer in five studies, in Huang G K 2016 (42), however, the main types of cancer were ovarian cancer, endometrial cancer, and carcinoma of the fallopian tube, which may cause potential confounding. Criteria for hospital discharge were reported in Nanthiphatthanachai 2020 (3) and Ertas 2013 (40), while others did not, which may influence the length of hospitalization and the result of heterogeneity.

### 4.3 Strengths and limitations

To our knowledge, this systematic review is one of the few to examine the effect of gum-chewing on the incidence of postoperative ileus after gynecological cancer surgery. In previous meta-analyses, gum-chewing was a major confounder, but in our review, the frequency and duration of gum-chewing were consistent across studies, reducing the risk of implementation bias. We chose postoperative ileus as the primary outcome of interest because it is more objective than time to first flatus. In addition, all of the trials included in our review were randomized controlled trials, which provide more reliable evidence of the association between gum-chewing and the incidence of postoperative ileus.

There are several limitations of this review that should be considered. Firstly, the number of trials included is relatively small (six RCTs), and more robust RCTs are needed to reach a conclusive result. Secondly, gum-chewing interventions were implemented in three different countries, which may lead to differences in the routine care received by control group participants. Finally, it seemed challenging to blind both outcome assessors and participants, which may have introduced detection and performance bias. Considering the potential influence of ethnicity or sociodemographic factors between different countries, the extrapolation of our results needs to be done with caution. We believe the influence could be minimal because gum chewing is a harmless practice and widely accepted in countries around the world.

## 5 Conclusions

In conclusion, this systematic review and meta-analysis of six RCTs suggests that gum-chewing is associated with early recovery of gastrointestinal function after gynecological cancer surgery and may be an effective and safe intervention for preventing postoperative ileus. Given the limited number of studies included in our analysis, multi-center studies with larger sample sizes and well-designed RCTs are warranted to assess the efficacy of gum-chewing on gastrointestinal function after gynecological cancer surgery.

## Data availability statement

The original contributions presented in the study are included in the article/supplementary material. Further inquiries can be directed to the corresponding author.

## Author contributions

Y-NY was involved in the conception design, literature review, and contributed to the first draft. HX was involved in the literature review, J-HR was involved in the consultation and participated in the data collection and analysis. N-JJ was involved in data analysis and interpretation, LD revised the manuscript and approved the final version. All authors contributed to the article and approved the submitted version.
